# Alginate oligosaccharides trigger multiple defense responses in tobacco and induce resistance to *Phytophthora infestans*

**DOI:** 10.3389/fpls.2025.1506873

**Published:** 2025-02-12

**Authors:** Chune Peng, Wei Xu, Xipan Wang, Fanxiao Meng, Yumeng Zhao, Qingbin Wang, Xinkun Wang, Rathna Silviya Lodi, Xiaodan Dong, Changxiang Zhu, Lizeng Peng

**Affiliations:** ^1^ Key Laboratory of Agro-Products Processing Technology of Shandong Province, Key Laboratory of Novel Food Resources Processing Ministry of Agriculture, Institute of Food and Nutrition Science and Technology, Shandong Academy of Agricultural Sciences, Jinan, China; ^2^ State Key Laboratory of Crop Biology, College of Life Sciences, Shandong Agricultural University, Tai’an, Shandong, China; ^3^ School of Life Sciences, Qilu Normal University, Jinan, China

**Keywords:** alginate oligosaccharides, immune inducer, defense response, salicylic acid, chitin elicitor receptor kinase

## Abstract

Alginate oligosaccharides (AOSs), important plant immunity inducers, are widely used in agriculture because of their important role in the biological control of crop diseases. However, the mechanism by which AOSs induce plant resistance to pathogens is not clear. Here, we report AOS with a degree of polymerization of 2–5, which was obtained by a newly reported enzyme Aly2. AOS treatment exhibited high activity in enhancing resistance to *Phytophthora infestans* (*P*. *infestans*). AOS significantly induced reactive oxygen species (ROS) accumulation, calcium influx, stomata closure, and callose deposition. The salicylic acid (SA) synthesis-related gene and the defense-related genes were upregulated after AOS treatment. A transcriptome file generated from AOS-treated seedlings verified the SA pathway and suggested the presence of chitin elicitor receptor kinase (CERK). The subsequent results showed that AtCERK1 binds AOS tightly, suggesting that AtCERK1 is responsible for AOS recognition. This study laid a theoretical foundation for the broad application of AOS.

## Introduction

During growth, plants are affected by many kinds of stresses, which can alter many plant processes. Plant diseases cause immense annual losses in crop yield, posing a great threat to food production. Approximately 3 billion kg of chemical reagents are used worldwide annually (Hernández et al., 2013), but approximately 1% of pesticides are effectively used on plants (Bernardes et al., 2015). The extensive use of agrochemicals has caused pernicious pesticide residues and environmental and soil pollution (Qin et al., 2014; Rodriguez-Salus et al., 2016; Xue et al., 2006), decreased crop quality, and threatened human health (Al-Wabel et al., 2016; Duan et al., 2008; Lozowicka et al., 2015; Yadav et al., 2015). Therefore, new strategies are urgently required to improve plants’ immunity and enhance their resistance to pathogens.

Plant immunity inducers are biological agents that can reduce the use of chemical pesticides while improving resistance to pathogens (Li et al., 2018a; Nisa et al., 2015). In addition to inducing the deposition of lignin, strengthening plant cell walls, and forming a physical barrier, such as callose that are deposited around the cell wall and plasmodesmata (Ton et al., 2009), to resist pathogen infection, plant immunity inducers also promote the production of endogenous substances such as pathogenesis-related proteins (PRs), reactive oxygen species (ROS), and salicylic acid (SA) (Cai et al., 2020; Jiang et al., 2023; Peng et al., 2020; Sticher et al., 1997). The non-expressor of PR (NPR) is a regulatory protein, and NPR1 plays a key role in this process (Durrant and Dong, 2004; Sun et al., 2018). PR-1A and NPR are the most abundant proteins produced by plants in response to pathogens via the SA pathway (Breen et al., 2017). When plant cells detect the presence of pathogens through recognition receptors on their surfaces, the phytohormone, such as the SA signaling pathway is activated, leading to the upregulation of the expression of the defense genes *PR-1a* and *NPR1*. This, in turn, induces resistance to pathogens.

Protein kinases on the cell surface are critical for transiting signals from the outside to the inside of the cell. From extracellular stimulation to the corresponding biological effect in cells, mitogen-activated protein kinase (MAPK) cascades must be activated. The MAPK pathway also affects plant resistance to pathogens by regulating stomatal closure. Oligosaccharides, such as mannan oligosaccharides (MOSs) and oligogalacturonic acid (OGA), also cause Ca^2+^ concentration changes and stomatal closure. The transient increase in Ca^2+^ and the MAPK cascade are important signaling pathways that stimulate the early defense response in plants (Lecourieux et al., 2006). NtMEK2 in the MAPK cascade pathway can be activated by multiple activators in tobacco and regulate phenylalanine lyase (PAL) expression, a key enzyme in SA synthesis (Yang et al., 2001). In addition, SA is an important hormone-signaling molecule that leads to plant systemic-acquired resistance.

Oligosaccharides are important plant immunity inducers that can be developed as a biostimulant. As a biological immunity inducer, AOS can be prepared through the enzymatic degradation of alginate and possesses advantages, such as low molecular weight, good water solubility, easy absorption, and non-polluting. AOS application in agriculture has become a popular research topic (Peng et al., 2018). Increasing evidence confirms the function of AOS in enhancing plant stress resistance, growth, and development (Hien et al., 2000; Hu et al., 2004; Iwasaki and Matsubara, 2000; Ma et al., 2010; Natsume et al., 1994; Tang et al., 2011). Studies have shown that AOS can promote root development and elongation in Komatsu (Yonemoto et al., 1993), barley (Tomoda et al., 1994), rice (Zhang et al., 2014), and carrot (Xing et al., 2020; Xu et al., 2003); reduce the damage caused by salt stress (Liu et al., 2013; Tang et al., 2011); enhance the tolerance of cucumber to water stress (Li et al., 2018b); enhance drought resistance in wheat through the SA pathway (Liu et al., 2013); and enhance the resistance to *Pseudomonas syringae* pv. *tomato* (*Pst*) DC3000 through the salicylic acid pathway (Zhang et al., 2019). However, relatively few studies have investigated the mechanism by which AOS induces plant resistance to pathogens, especially late blight of potato and tomato.

Oligosaccharides mimic the cell wall components of pathogens and are recognized by immune receptors/pattern recognition receptors on the plant cell surface to trigger pattern-triggered immunity (PTI), thereby enhancing plant resistance to disease (Jones and Dangl, 2006; Van Wees et al., 2008). Thus, lipopolysaccharide, chitin, OGA, and MOS (Zhang et al., 2019) can be recognized by plant receptors to stimulate PTI (Denoux et al., 2008; Hayafune et al., 2014; Yin et al., 2016). These elicitors are also known as pathogen-associated molecular patterns (PAMPs) and/or damage-associated molecular patterns (DAMPs) (Wiesel et al., 2014). Progress has been made in the studies of oligosaccharide receptors, such as chitin receptors (Espinoza et al., 2017; Liu et al., 2012) and OGA (Brutus et al., 2010). However, the receptor for AOS in plants has not been identified.

This study showed that AOS improved potato and tobacco resistance to *P. infestans*, and induced a series of defense responses in tobacco, including ROS accumulation, callose deposition, Ca^2+^ influx, and stomatal closure. Moreover, transcriptome sequencing was performed, and SA signal pathway-related genes were also detected. The receptor mutant *Arabidopsis thaliana* and enzyme-linked immunosorbent assays (ELISAs) were used to analyze the interaction between AOS, and the receptor was also analyzed. AOS can interact tightly with the cell surface receptor AtCERK1, while the binding ability was not significant between AOS and chitin elicitor binding protein (AtCEBiP), suggesting that AtCERK1 is the receptor of AOS in *Arabidopsis*. The results lay the foundation for the wide application of AOS as a new biopesticide.

## Materials and methods

### Materials and growth conditions

Sodium alginate was degraded by Aly2 for 12 h, and then AOS with a degree of polymerization (DP) of 2–5 was obtained (Peng et al., 2018) by using a Superdex 30 Increase 10/300 GL column, the mobile phase was 0.20 M NH_4_HCO_3_ at a flow rate of 0.4 mL/min, and the eluted fractions were monitored at 232 nm using a UV detector. In this study, wild-type *Nicotiana benthamiana* and wild-type *A. thaliana* were preserved and propagated. The plants *Solanum tuberosum* and *N*. *benthamiana* were grown at 22°C and 25°C, respectively, under 70% humidity with 16 h of light and 8 h of dark. *Arabidopsis thaliana* receptor mutants were cultured in an incubator (22°C, 16 h light/8 h dark). RNA extraction kits were purchased from Kangwei Reagent (Taizhou, China), reverse transcription kits were purchased from Hunan Aceri Bioengineering Co. (Changsha, China) and Novozymes (Beijing, China), and HRP-conjugated His_6_ was purchased from Sigma Aldrich (St. Louis, MO, USA). Biotin hydrazide, 2-(N-morpholino)-ethanesulfonic acid (MES), and 1-ethyl-3-(3-dimethylaminopropyl) carbodiimide (EDC) were purchased from Thermo Fisher Scientific (Waltham, MA, USA). *Escherichia coli* strains DH5α and BL21 (DE3) and *Pichia pastoris* were preserved in our laboratory. *Phytophthora infestans* strain was grown in the dark at 18°C using rye A agar. The T-DNA insertion mutants SALK_007193C for At3g21630 (*AtCERK1*) and SALK_206271C for At2g17120 (*AtCEBiP-LIKE1*) were obtained from EDITGENE Corporation (Guangzhou, China). All the other chemicals and reagents were of the highest quality. Primers were synthesized by Shanghai Shenggong Biotechnology Co. (Shanghai, China; **Table 1**).

**Table 1:** Primers used for sequencing in the present study.

Sequencing primers	Primer sequence (5'–3')
*Nb*-*actin*-F	5'-TTGGCTTACATTGCTCTTG-3'
*Nb*-*actin*-R	5'-TCATTGATGGTTGGAACAG-3'
*P*. *infestans*-O8-F	5'-GAAAGGCATAGAAGGTAGA-3'
*P*. *infestans*-O8-R	5'-TAACCGACCAAGTAGTAAA-3'
qRT-*NbSOD*-F	5'-GCAGCAGTGAAGGTGTTAGC-3'
qRT-*NbSOD*-R	5'-GGATTGTAATGTGGTCCCG-3'
qRT-*NbCAT*-F	5'-CACTCACCTTACCTGTGCTG-3'
qRT-*NbCAT*-R	5'-GAACTTCATTCCATCACGG-3'
qRT-*NbAPX*-F	5'-CATCAGGCTATTGGAACCC-3'
qRT-*NbAPX*-R	5'-GCTCTGTCTTGTCCTCTCTACC-3'
qRT-*NbRbohA*-F	5'-GAAGGCGGAGTTAAGGAGAT-3'
qRT-*NbRbohA*-R	5'-GAGCTCTATGAGCGCTGGAA-3'
qRT-*NbRbohB*-F	5'-GTGATGCTCGTTCTGCTCTT-3'
qRT-*NbRbohB*-R	5'-CTTTAGCCTCAGGGTGGTTG-3'
qRT-*NbICS*-F	5'-CAGTTGAAGAGCAGATAGAAG-3'
qRT-*NbICS*-R	5'-AAGTTCCATTGAAGCACATT-3'
qRT-*NbPAL*-F	5'-CTCAAGTTGCGGCTATTG-3'
qRT-*NbPAL*-R	5'-CATTCTTGGTCCTTCTATGTG-3'
qRT-*NbPR1a*-F	5'-CGTTGAGATGTGGGTCAATG-3'
qRT-*NbPR1a*-R	5'-CCTAGCACATCCAACACGAA-3'
qRT-*NbNPR1*-F	5'-GCACTTGAATCGGCTTAG-3'
qRT-*NbNPR1*-R	5'-TCTTCAGTTGACGCTCTT-3'
qRT-*AtCEBIP-LIKE1*-F	5'-GCTTGTTCCTCATCCGTCA-3'
qRT-*AtCEBIP-LIKE1*-R	5'-GCAAATGGCATTCTGACATCC-3'
qRT-*AtCERK1*-F	5'-GGAATTCCATATGAGGACTAGCTGTCCTTTAGC-3'
qRT-*AtCERK1*-R	5'-CCCAAGCTTAACAATTCACCAATACATT-3'

### *Phytophthora infestans* inoculation

Plates with *P*. *infestans* were flooded with 5 mL of ddH_2_O and scraped to release sporangia. The suspension was poured into a clean Petri dish, placed on ice, and stored at 4°C for 3 h to release zoospores. Then, the sporangia were counted and adjusted to 30,000 sporangia per milliliter. The potato leaves were inoculated with *P*. *infestans* (originally isolated from the province of Heilongjiang, north of China) at a concentration of 4 × 10^5^ sporangia mL^−1^. Droplets of 20 µL of *P. infestans* zoospores and sporangia suspension were added to the leaves of wild-type *N*. *benthamiana*, wild *Arabidopsis*, and *Arabidopsis* receptor mutants after AOS (100 μg/mL) treatment for 24 h. The leaves were placed in a plastic dish with ddH_2_O sprayed over them regularly. *Phytophthora infestans* infection was observed at 3 days post-inoculation (dpi), and *P. infestans* colonization was measured by quantitative real-time PCR. Briefly, total DNA was extracted from diseased leaves, including AOS-treated and untreated leaves, and then quantitative PCR was performed after DNA extraction by using the internal reference gene primers in **Table 1** (*Nb*-*actin* and *P*. *infestans-*O8 primer pairs). The expression levels of the internal reference gene of tobacco and *P. infestans* were measured to evaluate *P. infestans* accumulation. The primer sequences for the experiment are presented in **Table 1**.

The optimal working concentration of AOS was determined by spraying 0, 25, 50, 100, or 200 μg/mL of gradient AOS aqueous solution on potato Désirée/Eshu 3 leaves for 24 h, followed by inoculation with *P*. *infestans*; the other conditions were the same as those described above. There were three biological replicates for each treated sample.

### Histochemical staining of reactive oxygen species

Histochemical staining was performed using 3,3'-diaminobenzidinebutane (DAB) and nitroblue tetrazolium (NBT) to detect hydrogen peroxide (H_2_O_2_) and superoxide ion (O^2−^) accumulation, respectively, in the leaves of plants subjected to AOS and H_2_O treatment. Plant tissues were placed in 1 mg/mL of DAB solution, vacuum-infiltrated for 30 min, washed three times with deionized water, and reacted with H_2_O_2_ for 12–24 h under light at 28°C. The excess dye solution was washed away by using a boiled solution (ethanol:lactic acid:glycerin = 3:1:1) at 100°C for 10 min, and the leaves were imaged. For NBT staining, *N. benthamiana* leaves were immersed in 1% (M/V) sodium azide solution, which increases the permeability of cells, vacuum-immersed for 30 min, and then transferred to 0.5 mg/mL of NBT solution, followed by vacuum infiltration for 30 min. O^2−^ reacted with NBT to form a deep blue insoluble complex. The boiled solution (ethanol:lactic acid:glycerin = 3:1:1) was also used to wash off the excess dye solution, after which the leaves were imaged. The strengths of H_2_O_2_ and O^2−^ were quantitatively analyzed by ImageJ software.

For aniline blue staining, 20 mL of lactic acid, 20 mL of phenol, 40 mL of 20% glycerol, and 20 mL of deionized water were mixed evenly; the volume was adjusted to 100 mL; and then anhydrous ethanol was added at a volume ratio of ~2:1. *Nicotiana benthamiana* leaves were put into the above solution under vacuum for 30 min and then treated at 60°C for 30 min. The 0.01% aniline blue solution was added after washing with deionized water, and the leaves were kept at room temperature overnight without light. Finally, the leaves were preserved in 50% glycerol and were observed and photographed under a fluorescence microscope.

### Measurement of Ca^2+^ in guard cells and stomatal aperture measurement

*Nicotiana benthamiana* leaf epidermis strips were soaked in MES, pH 6.0 buffer under light for 3 h to open the stomata, and then a final concentration of 20 µmol/L of Fluo-3AM was added at 4°C for 2.5 h. The excess fluorescent dye was washed by using the MES buffer and then kept at room temperature for 1 h. The epidermis strips were treated with ddH_2_O and 100 μg/mL of AOS for 24 h, and then fluorescence was observed using a confocal microscope. Fluo-3AM was used to analyze Ca^2+^ accumulation in the guard cells. Each treatment included an investigation of at least three epidermis strips, and the experiment was repeated three times. Images of the stomatal aperture were captured with an Olympus BX43 microscope (Olympus, Tokyo, Japan) using the cellSens Standard software, and the diameters of 50 randomly selected stomata were measured. Each assay was repeated three times.

### RNA extraction and quantitative real-time PCR

Total RNA was extracted from tobacco leaves with TRIzol reagent (TaKaRa, Shiga, Japan) according to the manufacturer’s instructions. The cDNA was synthesized from 1 μg of total RNA using a FastKing gDNA Dispelling RT SuperMix kit (Tianjin, Beijing, China). Quantitative real-time PCR (qRT-PCR) was performed by using a Talent SYBR Green Kit (Tianjin, Beijing, China). Each reaction was conducted in triplicate and repeated three times. Bio-Rad CFX Manager software (Bio-Rad, California, USA) was used to analyze the data. The relative expression levels of the ROS-scavenging enzymes catalase (CAT), superoxide dismutase (SOD), ascorbate peroxidase (APX), and ROS-generating-related genes, including the respiratory burst oxidase homolog genes (*RbohA* and *RbohB*), were measured by qRT-PCR after AOS treatment for 0, 2, 4, 8, 12, and 24 h, respectively. Primer sequences for the experiment are presented in **Table 1**.

### Data analysis of RNA sequencing

The transcriptome was sequenced by Shanghai OE Biotech Co., Ltd. (Shanghai, China). RNA samples were taken from five- or six-leaf-stage leaves treated with H_2_O (0 h) or 100 μg/mL of AOS for 24 h. Each sample was analyzed three times. The samples were selected depending on quality (RIN score ≥ 7). All differential gene expression data were based on the following criteria: an absolute log_2_ ratio ≥1 and an FDR ≤0.001.

### Expression and purification of AtCERK1 and AtCEBiP-LIKE1

The two PCR products of the *AtCERK1* gene and the *AtCEBiP-LIKE1* gene were individually cloned into the pET-30a (+) vector and SacI vector. The proteins AtCERK1 (extracellular domain, At3g21630) and AtCEBiP-LIKE1 (At2g17120) were expressed by using BL21(DE3) and yeast with a His_6_ tag at the C-terminus, respectively. For AtCERK1 expression, *E. coli* cells harboring the recombinant plasmid were initially cultured in LB broth. When the cell density reached an OD_600_ of 0.8–1.0, the broth was supplemented with the inducer isopropyl 1-thio-*β*-D-galactopyranoside at a final concentration of 0.05 mM to initiate the expression of AtCERK1. AtCEBiP-LIKE1 was expressed in a similar manner, and yeast cells harboring AtCEBiP-LIKE1 were cultured in BMGY broth and then in BMMY broth. The broth was supplemented with 1% methanol to induce AtCEBiP-LIKE1 expression. Then, the proteins were purified by Ni^2+^ chelation chromatography according to the method provided by Peng et al. (2018).

### Interaction analysis

Molecular interactions were analyzed by using biotin-labeled AOS and CERK1/CEBiP-LIKE1 based on the ELISA method. AOS (8 mg/mL) was biotinylated in 0.1 M of MES (Sigma-Aldrich) (pH 5.5) biotin LC-hydrazide solution (Deepa et al., 2002; Peng et al., 2021). EDC (1 mg) was added to the reaction mixtures and reacted overnight at room temperature, and each reaction mixture was desalted three times with PBS and centrifuged (4°C, 2,000 rpm, 2 min) to obtain the biotin-labeled AOS. Then, 50 μL of 1 mg/mL streptavidin was added to a 96-well plate and sealed at 4°C overnight, 1% BSA was added for 1 h, and biotin-labeled AOS was added at room temperature for 2 h. The binding reaction of chitin-binding proteins (AtCERK1 and AtCEBiP-LIKE1) was carried out at 4°C overnight, and the above process was avoided from light and washed with PBS. Then, the antibody was added for 30 min, the sample was washed with PBST, TMB was used to develop the color, and the absorbance was measured at 450 nm.

## Results

### AOS protected plants against *Phytophthora infestans* infection

**Figure 1:**
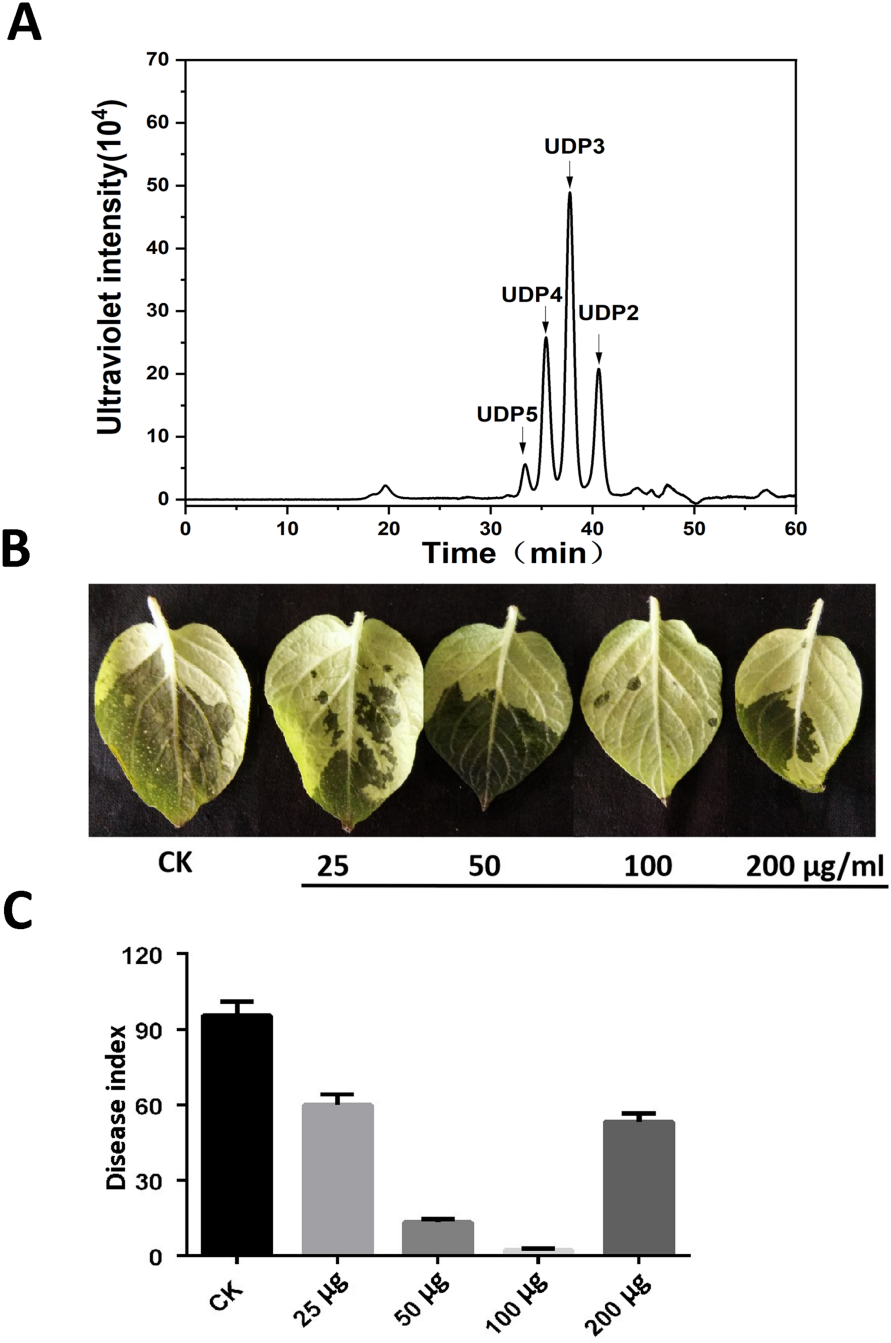
AOS treatment enhanced potato resistance against *Phytophthora infestans*. **(A)** Approximately 50 μg of AOS with a degree of polymerization (DP) of 2–5 was loaded on a Superdex 30 Increase 10/300 GL column. The elution positions of the unsaturated oligosaccharide product fractions with different degrees of polymerization are indicated by arrows: UDP2, unsaturated disaccharide; UDP3, unsaturated trisaccharide; UDP4, unsaturated tetrasaccharide; UDP5, unsaturated pentasaccharide. **(B)** Wild-type Désirée was inoculated with *P*. *infestans* after a gradient concentration (0, 25, 50, 100, and 200 μg/mL) of AOS treatment for 24 h, and the phenotype was observed at 3 dpi. **(C)** The disease index of *P*. *infestans* at 3 dpi. Error bars show the mean ± SD of three replicates (at least 20 plants per replicate).

The potato cultivar *Désirée* and *P*. *infestan*s were used as materials to examine the activity of AOS with a DP of 2–5 (**Figure 1A**) against late blight. The potato leaves were inoculated with *P*. *infestans* after being sprayed with different concentrations of AOS for 24 h at 0, 25, 50, 100, and 200 μg/mL. Images were photographed at 4 dpi, and AOS could significantly enhance the resistance of potato to late blight. The infected area and disease index was gradually decreasing with increasing AOS concentration, but it was greater when the concentration was increased to 200 μg/mL (**Figures 1B, C**). We hence used 100 μg/mL of AOS in the next experiment.

### AOS promoted hydrogen peroxide accumulation

**Figure 2:**
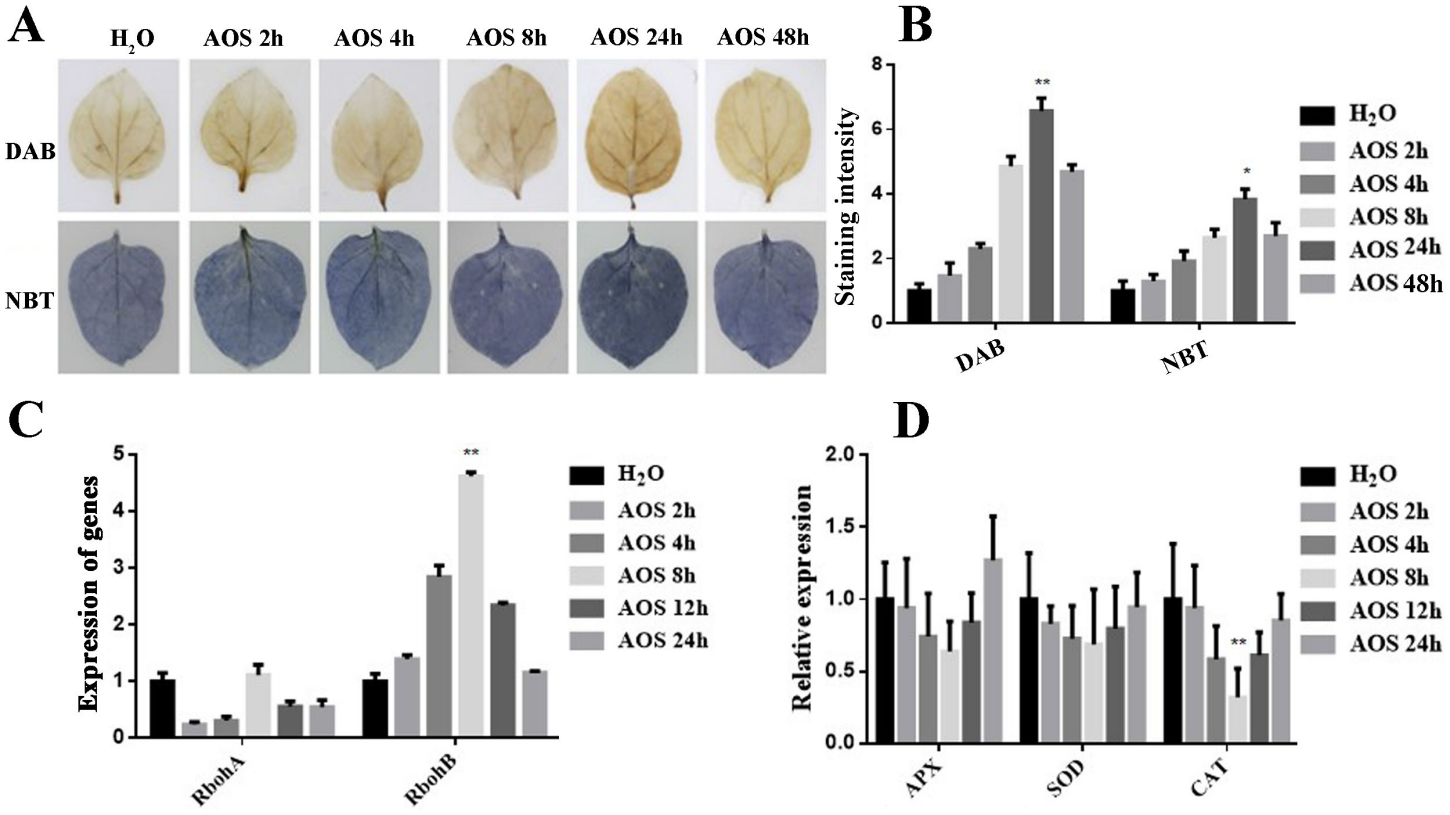
AOS promoted hydrogen peroxide and superoxide anion accumulation. AOS promoted hydrogen peroxide accumulation in *Nicotiana benthamiana.* **(A)** Hydrogen peroxide (up) and superoxide accumulation (down) were measured in the leaves treated with 100 μg/mL of AOS at different (0, 2, 4, 8, 24, 48) hours post-treatment (hpt) (*n* = 6). **(B)** Quantification of hydrogen peroxide and superoxide levels in *N. benthamiana* treated with 100 μg/mL of AOS at 0, 2, 4, 8, 12, and 24 hpt. Data are shown as the mean (*n* = 6) ± SD. **(C)** qRT-PCR analysis of *RbohA* and *RbohB* expression at various time intervals. **(D)** CAT, SOD, and APX were detected using qRT-PCR at various time intervals. Data are shown as the mean (*n* = 6) ± SD. * indicates significant differences determined using the Student’s *t*-test (*p* < 0.05), and ** indicates extremely significant differences determined using the Student’s *t*-test (*p* < 0.01).

To investigate whether AOS regulates ROS accumulation, DAB and NBT staining were applied to evaluate the H_2_O_2_ and O^2−^ levels in tobacco leaves that were detached from the water-spraying group and the 100-μg/mL AOS-spraying group. **Figures 2A, B** show that DAB staining was first strengthened and then weakened with the extension of time. DAB staining was the deepest after 24 h of spraying AOS, which indicated that the accumulation of H_2_O_2_ was elevated. Similarly, NBT staining was the deepest after 24 h of spraying AOS, suggesting that AOS could also promote O^2−^ accumulation in plants.

The mRNA levels of several important genes encoding the ROS-scavenging enzymes CAT, SOD, APX, and ROS-generating-related genes, including the respiratory burst oxidase homolog genes (*RbohA* and *RbohB*), were determined by qRT-PCR analysis and monitored before and after AOS treatment. After AOS treatment, there was a significant increase in the expression level of the ROS generation-related gene *RbohB* (**Figure 2C**). Conversely, the expression level of the *CAT* gene decreased significantly, while there was no significant change in the expression levels of the *SOD* and *APX* genes (**Figure 2D**). These results suggest that AOS may enhance hydrogen peroxide accumulation by inhibiting *CAT* gene expression and promoting *RbohB* gene expression.

### AOS with a DP of 2–5 leads to Ca^2+^ influx

**Figure 3:**
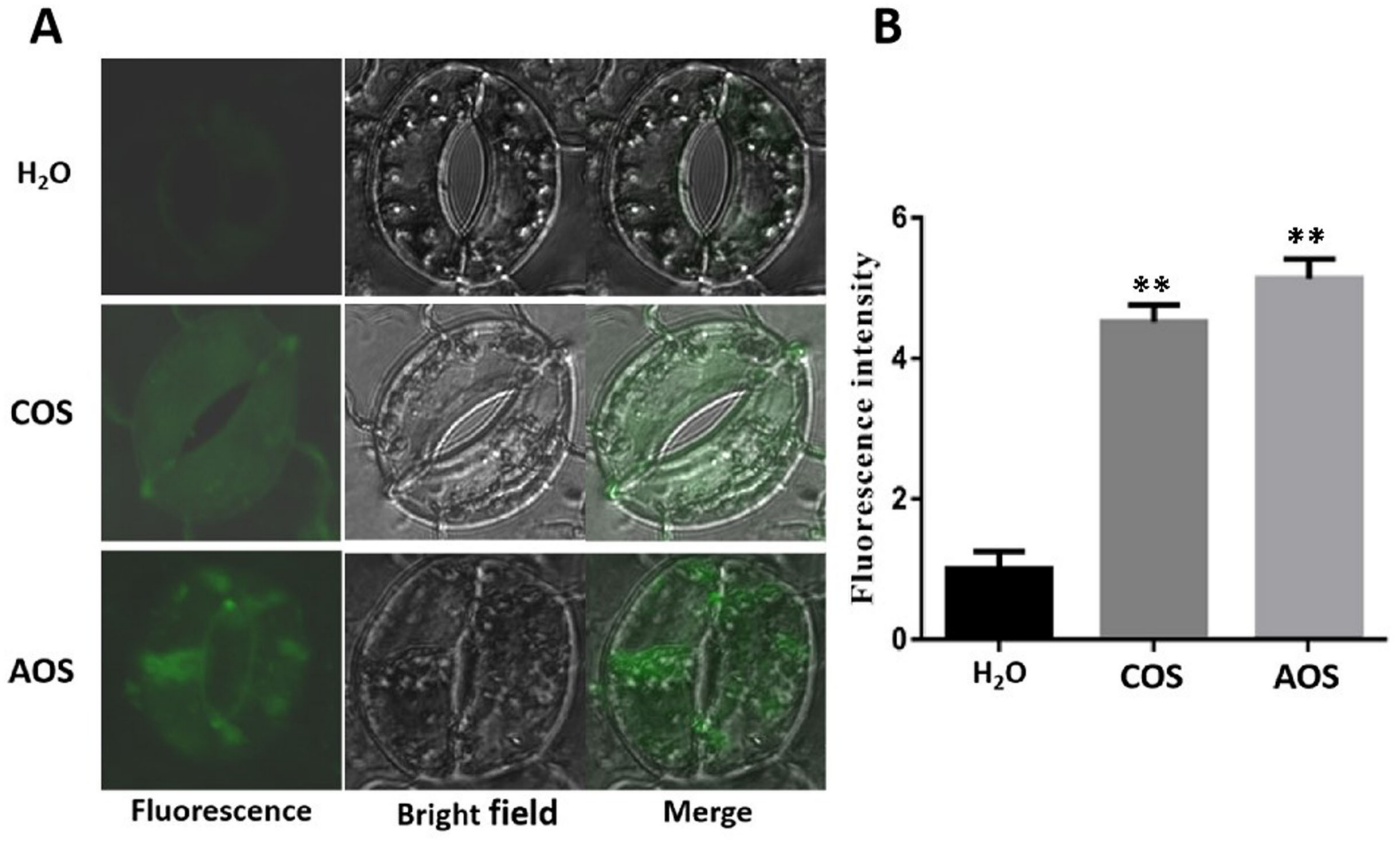
AOS promotes intracellular Ca^2+^ accumulation in the guard cells and stomatal closure of *Nicotiana benthamiana*. The calcium-specific fluorescence probe Fluo-3AM was preincubated with epidermal peels at 4°C and then kept at room temperature for 1h. The fluorescence was observed by a laser confocal microscope after incubation with H_2_O, COS (1,000 μg/mL), and AOS (100 μg/mL) for 3h. **(A)** Representative images (enlarged images). **(B)** Quantitative analysis of Ca^2+^ concentration by using the ZEN software. The experiments were repeated three times. Error bars indicate SEM. Statistics by the Student’s *t*-test (**p ≤ 0.01).

Ca^2+^ is an important secondary messenger that triggers plant defense response. Ca^2+^ usually stays at a low concentration in the plant cell cytoplasm, whereas biotic stresses, pathogen infection, and elicitor promote Ca^2+^ influx from the extracellular to the cytoplasm, thus leading to a rapid transient cytoplasmic Ca^2+^ increase (Zang et al., 2019). To investigate whether AOS leads to Ca^2+^ influx, the fluorescent-labeled Fluo-3AM was used to evaluate the cytoplasmic Ca^2+^ levels. ddH_2_O and COS were used as negative and positive controls, respectively (Zhang et al., 2009; Iriti and Varoni, 2015). There was no obvious fluorescence in the ddH_2_O-treated plant tissues, while the COS- and AOS-treated guard cells showed obvious fluorescence in the cells, indicating that AOS treatment significantly promoted Ca^2+^ influx in the guard cells. Moreover, AOS promoted stomatal closure, suggesting the prevention of pathogen infection (**Figure 3**).

To ascertain the ability of AOS to induce callose deposition in plants, wild-type *N. benthamiana* was subjected to 100 μg/mL of AOS spray treatment, while H_2_O was used as a control. Subsequently, aniline blue staining was conducted after 24 h. Microscopic examination (**Supplementary Figure S1A**) of the stained samples revealed conspicuous callose deposition surrounding the veins of *N. benthamiana* treated with AOS compared to the control group. The fluorescence intensity (**Supplementary Figure S1B**) was quantified and found to be consistent with the observed phenotype. These results indicate that AOS can effectively enhance callose deposition in plants and consequently improve their resistance against pathogen infections.

### AOS activated the SA signaling pathway

**Figure 4:**
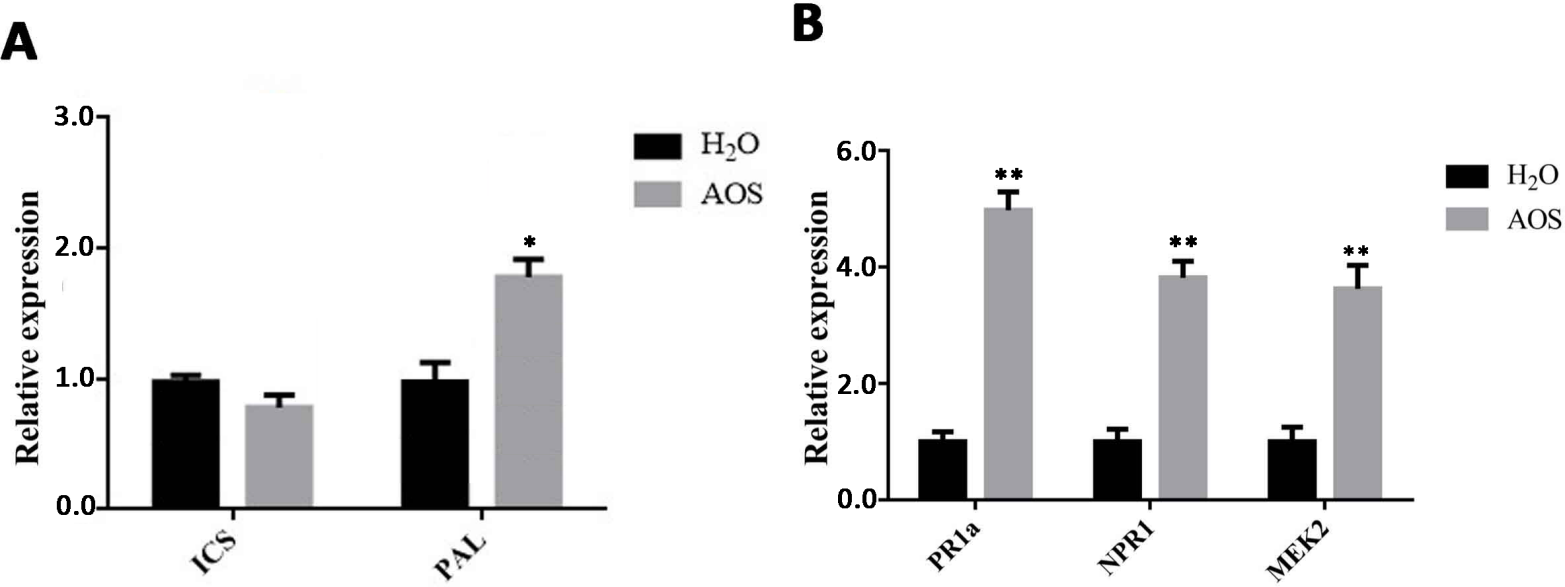
AOS promotes the accumulation of SA in *Nicotiana benthamiana*. **(A)** The SA biosynthesis-associated genes ICS1 and PAL were measured at 24 hpt using qRT-PCR (means ± SD, *n* ≥ 3). **(B)** The SA signaling-associated genes *PR1a* and *NPR1* and the *MEK2* gene were measured at 24 hpt using qRT-PCR (means ± SD, *n* ≥ 3). * indicates extremely significant differences determined using the Student’s *t*-test (*p* < 0.05); ** indicates extremely significant differences determined using the Student’s *t*-test (*p* < 0.01).

Plants synthesize SA mainly by the isochorismate synthase (ICS) and phenylalanine ammonia lyase (PAL) pathways. Thus, we detected the gene expression level of the *ICS* and *PAL* genes by using qRT-PCR. The results showed that the *PAL* gene was upregulated by AOS, whereas the *ICS* gene basically remained unchanged (**Figure 4A**). These results suggested that AOS promotes SA synthesis by improving the *PAL* transcription level.

Pathogenesis-related (PR) proteins play an important role in plant defense. They can improve plant disease resistance by inhibiting pathogen reproduction and are mainly involved in plant-acquired systemic resistance. PR-1A and NPR are the key factors in the SA pathway, involved in *N*. *benthamiana*’s resistance to pathogens or other pathogens, such as *Phytophthora* (Zang et al., 2019). The expression levels of genes related to these signaling pathways were examined by qRT-PCR in *N. benthamiana* leaves after AOS treatment for 24 h. The expression levels of the *PR1a* and *NPR1* genes, key genes in the SA pathway, were upregulated significantly (**Figure 4B**). This suggests that AOS can promote the expression of the NPR1 and PR1 proteins via the SA signaling pathway, enabling plants to acquire systemic resistance and enhance their resistance to late blight.

The MEK2 (MAPK kinase)-SIPK/WIPK cascade, an *N*. *benthamiana* mitogen-activated protein kinase (MAPK) cascade, is an essential signaling pathway for plant immunity and is involved in the hypersensitive response (HR) accompanied by cell death. **Figure 4B** shows that *MEK2* genes were also upregulated significantly, suggesting that AOS may activate plant immunity through the MAPK cascade.

### Quantitative differences in gene expression in *Nicotiana benthamiana* after AOS treatment

**Figure 5:**
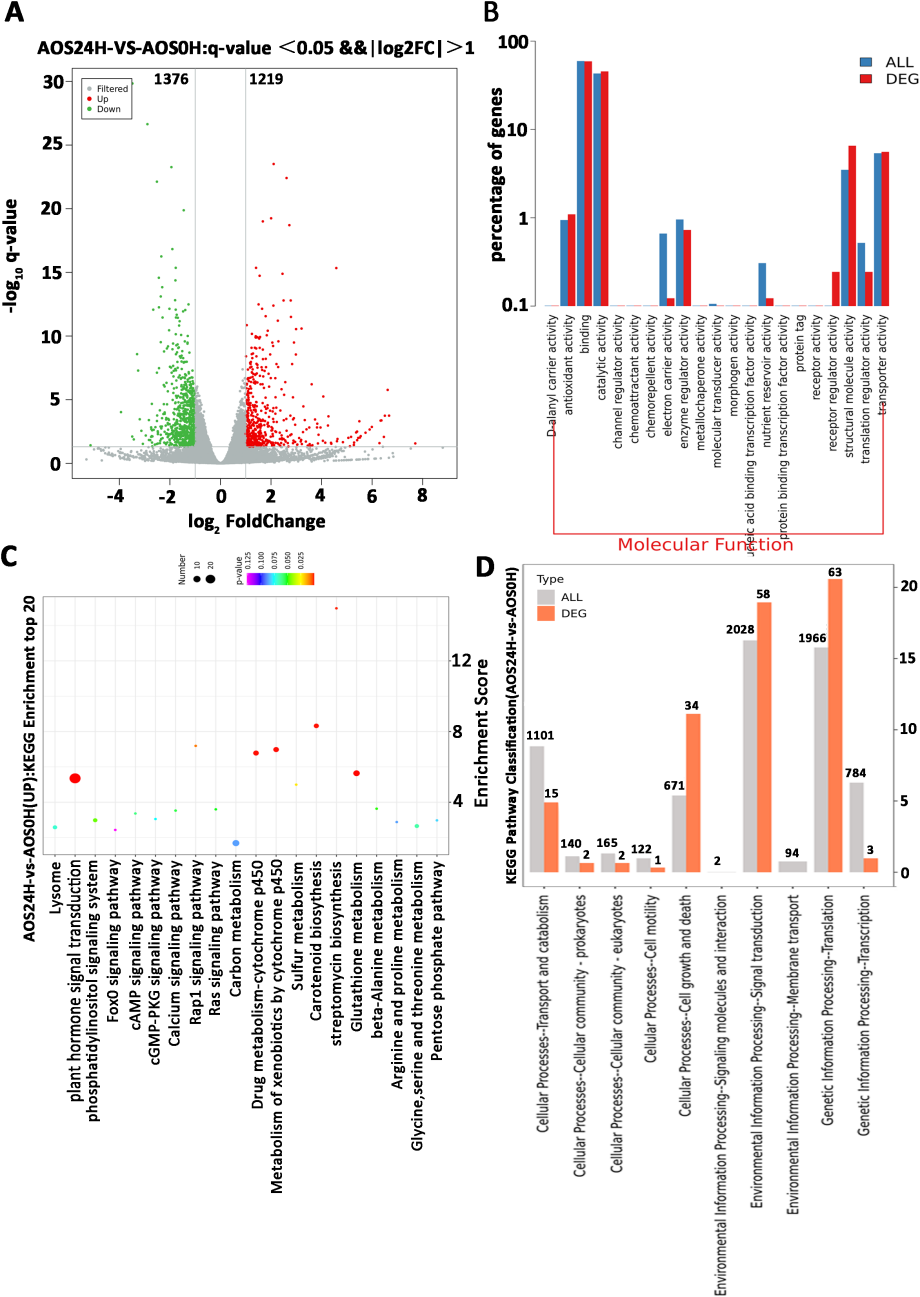
A parametric transcriptome analysis after AOS treatment in *Nicotiana benthamiana*. **(A)** Volcano plot showing the fold change and adjusted *p*-value of the normalized read counts of the transcriptome sequencing data. The criteria of log2| (fold change)≥1 and padj ≤0.05 were used to identify the DEGs. The green dots indicate the downregulated DEGs, and the red dots indicate the upregulated DEGs. **(B)** Comparative plots of the distribution of DEGs and all genes at the GO level 2. The horizontal axis is the name of the entry, and the vertical axis indicates the number of genes corresponding to the entry and their percentages. **(C)** The characteristic KEGG pathways with significant enrichment of DEGs after treatment with AOS. **(D)** Partial KEGG pathway classification. The abscissa axis is the ratio of DEGs in a pathway: all DEGs in the KEGG level 2 pathway (%); the ordinate axis is the name of the pathway. The numbers above the column represent the quantity of DEGs in the pathway.

Transcriptome sequencing analysis was performed on the leaves treated with AOS to reveal the role of AOS in inducing plant resistance. The leaves were harvested after 24 h of 100 μg/mL AOS treatment. The differentially expressed genes (DEGs) after AOS and H_2_O treatment (AOS0H) were analyzed. The results revealed 2,595 DEGs in the AOS24H group vs. the AOS0H group, of which 1,219 genes were upregulated and 1,376 genes were downregulated (**Figure 5A**).

Most of the AOS-regulated genes at 24 hpt were annotated with a wide range of Gene Ontology (GO) terms in the molecular functions (**Figure 5B**). Molecular function results suggested receptor activity and receptor regulator activity, and the transcriptome DEG showed that the CERK1 gene expression level was upregulated, consistent with the results in **Supplementary Figure S2B**. The enriched GO terms (**Table 2**) of the biological process category included positive regulation of defense response, chitinase activity, and signal transduction. Within the KEGG classification, plant hormone signal transduction genes showed the greatest changes in expression (**Figure 5C**), and 155 DEGs were upregulated (**Figure 5D**). These results further verified that resistance to *P*. *infestans* was induced by the SA signaling pathway. Plant chitinases are described as pathogen-associated proteins because they are induced in response to invasion by plant pathogens. The genes related to the chitin catabolic process and chitinase (Niben101Scf01789g03003, Niben101Scf02041g00002, and Niben101Scf02171g00007) were also significantly upregulated (**Table 2**). Based on the above results, we speculate that the receptors in the plants of AOS may be related to chitin elicitor receptor proteins.

**Table 2:** Gene Ontology (GO) enrichment of upregulated genes.

GO accession	Description (term)	DEG number	Category
GO:0004568	Chitinase activity	3	Molecular_function
GO:0006032	Chitin catabolic process	3	Biological_process
GO:0035556	Intracellular signal transduction	7	Biological_process
GO:0008152	Metabolic process	67	Biological_process
GO:0009725	Response to hormone	4	Biological_process
GO:0005388	Calcium-transporting ATPase activity	2	Molecular_function
GO:0070588	Calcium ion transmembrane transport	2	Biological_process
GO:0005047	Signal recognition particle binding	1	Molecular_function
GO:0008061	Chitin binding	1	Molecular_function
GO:0019901	Protein kinase binding	2	Molecular_function
GO:0009966	Regulation of signal transduction	1	Biological_process
GO:0007165	Signal transduction	10	Biological_process
GO:0004871	Signal transducer activity	3	Molecular_function
GO:0006952	Defense response	2	Biological_process

To determine the AOS receptor protein in plants, several *Arabidopsis* receptor loss mutants, including the T-DNA insertion mutants for At3g21630 (*AtCERK1*) and At2g17120 (*AtCEBiP-LIKE1*), were inoculated with *P. infestans*. There was no significant difference in mortality in the *Arabidopsis* mutant group after AOS or ddH_2_O treatment, while AOS improved the resistance to *P. infestans* in the Columbia wild-type *Arabidopsis* group (**Supplementary Figure S2A**). The levels of the *CEBiP-LIKE1* gene and the *AtCERK1* gene were significantly increased after AOS treatment for 24 h (**Supplementary Figure S2B**).

**Figure 6:**
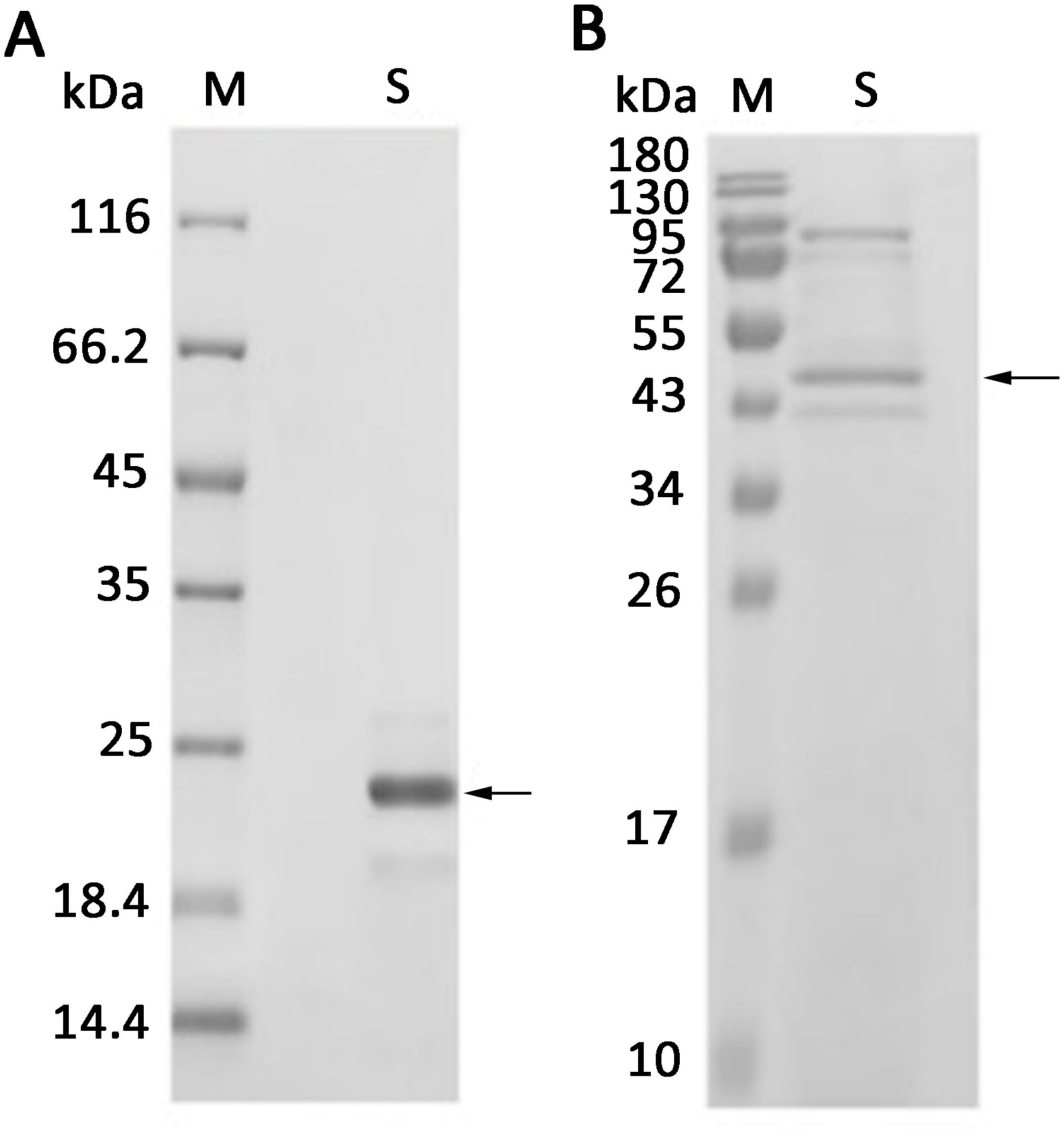
Expression of AtCERK1 and AtCEBiP-LIKE1 protein. **(A)** AtCERK1 was overexpressed in *Escherichia coli* BL21 (DE3) and was assessed by SDS-PAGE using 12% (w/v) polyacrylamide gels, followed by staining with Coomassie Brilliant Blue. M: unstained protein molecular weight marker SM0431; S: AtCERK1 protein purified from the *E. coli* supernatant. **(B)** AtCEBiP-LIKE1 was overexpressed in yeast and was assessed by SDS-PAGE. M: stained protein molecular weight marker PageRuler; S: AtCEBiP-LIKE1 protein purified from the yeast cell lysate.

To analyze the interaction between AOS and the receptor proteins, the receptors AtCERK1 (extracellular domain, At3g21630) and AtCEBiP-LIKE1 (At2g17120) were expressed and purified. SDS-PAGE showed that the molecular weights of AtCERK1 (with amino acids ranging from 26 to 230) with a His_6_ tag at the C-terminus and AtCEBiP-LIKE1 (with a secretory peptide at the N-terminus and a His_6_ tag at the C-terminus) were approximately 23 kDa (**Figure 6A**) and 47 kDa (**Figure 6B**), respectively, which are consistent with the theoretical molecular weights.

**Figure 7:**
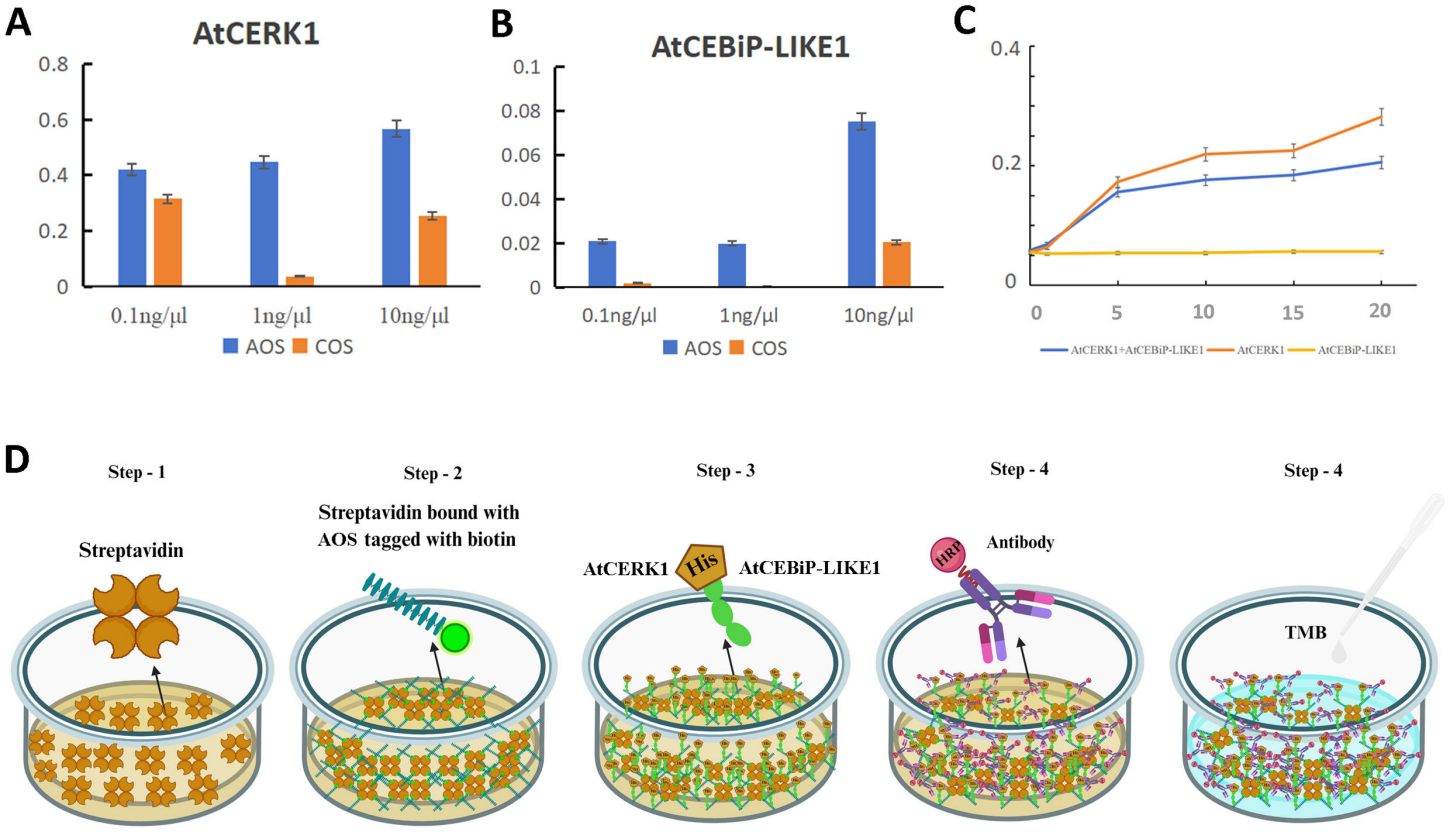
AtCERK1 is a key receptor kinase when AOS induces resistance to pathogens. Absorbance values at 450 nm for AOS and 0.1, 1.0, and 10 ng/μL of AtCERK1 **(A)** and AtCEBiP-LIKE1 **(B)**. COS was used as a control. **(C)** Association curve of the different concentrations of AtCERK1 and AtCEBiP-LIKE1. **(D)** Schematic representation of the binding assay of AOS with AtCERK1 or AtCEBiP-LIKE1. Step 1: streptavidin plate incubation; step 2: addition of biotin-labeled AOS; step 3: addition of AtCERK1/AtCEBiP-LIKE1 protein with a His tag; step 4: addition of HRP-labeled anti-His antibody; step 5: TMB chromatography at 450 nm. A high reading with a dark yellow color indicates a high degree of binding.

Then, ELISA was used to analyze the interaction between the biotin-labeled AOS and the different concentrations of AtCEBiP-LIKE1, as well as between the biotin-labeled AOS and the different concentrations of AtCERK1. The OD_450_ absorption value was greatest (0.60) when the AtCERK1 concentration was 1 ng/μL but was only 0.056 when the AtCEBiP-LIKE1 concentration was 1 ng/μL. The data suggested that AOS binds tightly to AtCERK1, while AOS cannot bind tightly to AtCEBiP-LIKE1. Furthermore, COS was used as a control and had a binding affinity to AtCERK1 and AtCEBiP-LIKE1, but the affinity was lower than that of AOS (**Figures 7A, B**). For further confirmation of AOS combined with CERK1, different concentrations of AtCERK1 and AtCEBiP-LIKE1 and a mix of AtCERK1 and AtCEBiP-LIKE1 were used to check the affinity to AOS. The results (**Figure 7C**) showed that with the increase of CERK1 concentration, the absorption value was higher to a certain extent; however, the concentration of AtCEBiP-LIKE1 had no significant effect on the absorption value. Identical molar values of AtCERK1 and AtCEBiP-LIKE1 were used to bind AOS, and the absorption value was lower when only AtCERK1 was used. The above results confirmed that AOS binds tightly to AtCERK1, indicating that AtCERK1 is the main plant receptor of AOS and is involved in the resistance induced by AOS. Moreover, the ELISA process and a schematic diagram of the interaction between AOS and AtCERK1/AtCEBiP-LIKE1 are shown in **Figure 7D**.

## Discussion

Since the 1960s, a large number of studies on oligosaccharide elicitors have been reported, with researchers concluding that oligosaccharides have certain biological activities, such as stimulating systemic responses and regulating plant growth and development, reproduction, and immunity (Jia et al., 2016; Salachna et al., 2018). Currently, the role of oligosaccharides in immunity has been intensively studied, and a variety of oligosaccharide products have been widely used. Oligosaccharides, such as COS, have been widely reported as PAMPs (Jia et al., 2016; Kim and Rajapakse, 2005). However, few studies have focused on the elicitor activities of AOS on plant immunity. The immune elicitor AOS against *P*. *infestation* was explored for the first time in this study.

AOSs (UDP2-UDP5) with specific structural characteristics have been prepared by using a new alginate lyase, Aly2 (Peng et al., 2018). The results of this study demonstrated that AOS improved plant resistance to *P*. *infestans* (**Figure 1**) and triggered various defense and resistance responses in tobacco, including increased ROS bursts (**Figure 2**), callose deposits (**Supplementary Figure S1**), intracellular Ca^2+^, stomatal closure (**Figure 3**), and defense-related gene expression (**Figure 4**). Moreover, transcriptome sequencing analysis revealed that AOS treatment upregulated the expression level of the genes of the phytohormone signaling pathway and the chitosan biosynthesis pathway, three of which were related to chitinase (**Figure 5**; **Table 2**), and these genes can promote plant resistance to pathogens. Chitinase is an extracellular complex of enzymes that degrade chitin and has the application value of hydrolyzing the cell wall of the pathogen fungi to inhibit growth. Chitinase can degrade chitin-producing N-acetylglucosamine oligomers or monomers, and the above oligomers can bind to the plant surface receptor CERK1 and stimulate plant disease resistance signals. Hence, the elicitor AOS identified in this study can be regarded as a novel PAMP.

Plants produce signal molecules when pathogens are recognized by cell surface receptors, and then the infection signal is transmitted to the cell through the signaling pathway, where it can cause local or systemic resistance (Baccelli et al., 2017). The phytohormone SA plays important roles in regulating disease resistance. In this study, the SA synthesis key gene *PAL* was upregulated (**Figure 4A**), and qRT-PCR revealed that the expression of the related marker genes *PR1A* and *NPR1* in the SA pathway increased (**Figure 4B**).

Many elicitors (Boller and Felix, 2009), including the elongation factor (Zipfel et al., 2006), flagellin (Denoux et al., 2008), chitin (Liu et al., 2016; Shinya et al., 2015), and other oligosaccharides (e.g., OGA) (Benedetti et al., 2015), can induce plant defense responses and improve plant resistance to pathogens. These elicitors are PAMPs, which can interact with the plant receptors to activate the PTI.

CERK1 is a plasma membrane protein that contains three LysM motifs in its extracellular domain and an intracellular Ser/Thr kinase domain with autophosphorylation/myelin basic protein kinase activity. It plays a key role in plants, detecting fungal microbe-related molecular patterns. Currently, it is regarded as a key receptor for plant immunity and symbiosis (Miya et al., 2007; Yang et al., 2022). CEBiP is a membrane glycoprotein with LysM motifs that functions as a cell surface receptor for chitin elicitors in rice (Liu et al., 2016) and plays an important role in the recognition of chitin. The AtCERK1 and AtCEBiP-LIKE1 proteins were expressed in this study (**Figure 6**), and the results showed that AtCERK1 binds AOS tightly, suggesting that AtCERK1 interacts with AOS, but this is not the case for AtCEBiP-LIKE1 and AOS (**Figure 7**). These results are consistent with those of Tomonori Shinya, who reported that AtCERK1 alone is sufficient for AOS detection (Shinya et al., 2012).

## Conclusion

We demonstrated that the elicitor AOS could induce plant resistance to late blight for the first time. The elicitor AOS activates the SA pathway and a series of defense responses to improve its resistance to pathogens. Moreover, AtCERK1 which binds to AOS is first reported here. We speculate that AOS is recognized by the receptor kinase CERK1 and transmits the signal to cells via its kinase activity and induces a series of defense responses, but the detailed signal pathway should be studied in-depth in the future. This study lays the theoretical foundation for AOS’s wide plant nosotropic applications.

## Data Availability

The Transcriptome Sequence data are deposited the in the NCBI Sequence Read Archive (SRA) under accession number PRJNA1097808. The datasets analyzed during the current study are available from the corresponding author on reasonable request.

## References

[B1] Al-Wabel M. El-Saeid M. H. El-Naggar A. H. Al-Romian F. A. Osman K. Elnazi K.. (2016). Spatial distribution of pesticide residues in the groundwater of a condensed agricultural area. Arab J. Geosci 9, 120. doi: 10.1007/s12517-015-2122-y

[B2] Baccelli I. Glauser G. Mauch-Mani B. (2017). The accumulation of β-aminobutyric acid is controlled by the plant’s immune system. Planta 246, 791–796. doi: 10.1007/s00425-017-2751-3 28762076

[B3] Benedetti M. Pontiggia D. Raggi S. Cheng Z. Scaloni F. Ferrari S.. (2015). Plant immunity triggered by engineered *in vivo* release of oligogalacturonides, damage-associated molecular patterns. Proc. Natl. Acad. Sci. U.S.A. 112, 5533–5538. doi: 10.1073/pnas.1504154112 25870275 PMC4418913

[B4] Bernardes M. F. F. Pazin M. Dorta L. C. P. Bernardes M. F. F. Pazin M. Dorta L. C. P. (2015). “Impact of pesticides on environmental and human health,” in Toxicology Studies - Cells, Drugs and Environment (Limited167-169 Great Portland Street,London, W1W 5PF, United Kingdom: IntechOpen). doi: 10.5772/59710

[B5] Boller T. Felix G. (2009). A renaissance of elicitors: perception of microbe-associated molecular patterns and danger signals by pattern-recognition receptors. Annu. Rev. Plant Biol. 60, 379–406. doi: 10.1146/annurev.arplant.57.032905.105346 19400727

[B6] Breen S. Williams S. J. Outram M. Kobe B. Solomon P. S. (2017). Emerging insights into the functions of pathogenesis-related protein 1. Trends Plant Sci. 22, 871–879. doi: 10.1016/j.tplants.2017.06.013 28743380

[B7] Brutus A. Sicilia F. Macone A. Cervone F. De Lorenzo G. (2010). A domain swap approach reveals a role of the plant wall-associated kinase 1 (WAK1) as a receptor of oligogalacturonides. Proc. Natl. Acad. Sci. U.S.A. 107, 9452–9457. doi: 10.1073/pnas.1000675107 20439716 PMC2889104

[B8] Cai Y. Yang T. Mitrano D. M. Heuberger M. Hufenus R. Nowack B. (2020). Systematic study of microplastic fiber release from 12 different polyester textiles during washing. Environ. Sci. Technol. 54, 4847–4855. doi: 10.1021/acs.est.9b07395 32250104

[B9] Deepa S. S. Umehara Y. Higashiyama S. Itoh N. Sugahara K. (2002). Specific Molecular Interactions of Oversulfated Chondroitin Sulfate E with Various Heparin-binding Growth Factors: inplications as a physiological binding partner in the brain and other tissues*. J. Biol. Chem. 277, 43707–43716. doi: 10.1074/jbc.M207105200 12221095

[B10] Denoux C. Galletti R. Mammarella N. Gopalan S. Werck D. De Lorenzo G.. (2008). Activation of defense response pathways by OGs and Flg22 elicitors in *Arabidopsis* seedlings. Mol. Plant 1, 423–445. doi: 10.1093/mp/ssn019 19825551 PMC2954645

[B11] Duan L. Zhang N. Wang Y. Zhang C. Zhu L. Chen W. (2008). Release of hexachlorocyclohexanes from historically and freshly contaminated soils in China: implications for fate and regulation. Environ. pollut. 156, 753–759. doi: 10.1016/j.envpol.2008.06.006 18635298

[B12] Durrant W. E. Dong X. (2004). Systemic acquired resistance. Annu. Rev. Phytopathol. 42, 185–209. doi: 10.1146/annurev.phyto.42.040803.140421 15283665

[B13] Espinoza C. Liang Y. Stacey G. (2017). Chitin receptor CERK1 links salt stress and chitin-triggered innate immunity in *Arabidopsis* . Plant J. 89, 984–995. doi: 10.1111/tpj.13437 27888535

[B14] Hayafune M. Berisio R. Marchetti R. Silipo A. Kayama M. Desaki Y.. (2014). Chitin-induced activation of immune signaling by the *rice* receptor CEBiP relies on a unique sandwich-type dimerization. Proc. Natl. Acad. Sci. U.S.A. 111, E404–E413. doi: 10.1073/pnas.1312099111 24395781 PMC3903257

[B15] Hernández A. F. Gil F. Lacasaña M. Rodríguez-Barranco M. Tsatsakis A. M. Requena M.. (2013). Pesticide exposure and genetic variation in xenobiotic-metabolizing enzymes interact to induce biochemical liver damage. Food Chem. Toxicol. 61, 144–151. doi: 10.1016/j.fct.2013.05.012 23688862

[B16] Hien N. Q. Nagasawa N. Tham L. X. Yoshii F. Dang V. H. Mitomo H.. (2000). Growth-promotion of plants with depolymerized alginates by irradiation. Radiat. Phys. Chem. 59, 97–101. doi: 10.1016/S0969-806X(99)00522-8

[B17] Hu X. Jiang X. Hwang H. Liu S. Guan H. (2004). Promotive effects of alginate-derived oligosaccharide on *maize* seed germination. J. Appl. Phycol 16, 73–76. doi: 10.1023/B:JAPH.0000019139.35046.0c

[B18] Iriti M. Varoni E. M. (2015). Chitosan-induced antiviral activity and innate immunity in plants. Environ. Sci. pollut. Res. Int. 22, 2935–2944. doi: 10.1007/s11356-014-3571-7 25226839

[B19] Iwasaki K. Matsubara Y. (2000). Purification of alginate oligosaccharides with root growth-promoting activity toward *lettuce* . Biosci. Biotechnol. Biochem. 64, 1067–1070. doi: 10.1271/bbb.64.1067 10879484

[B20] Jia X. Meng Q. Zeng H. Wang W. Yin H. (2016). Chitosan oligosaccharide induces resistance to Tobacco mosaic virus in *Arabidopsis* via the salicylic acid-mediated signalling pathway. Sci. Rep. 6, 26144. doi: 10.1038/srep26144 27189192 PMC4870575

[B21] Jiang X. Su H. Jiang J. H. Neelin J. D. Wu L. Tsushima Y.. (2023). Muted extratropical low cloud seasonal cycle is closely linked to underestimated climate sensitivity in models. Nat. Commun. 14, 5586. doi: 10.1038/s41467-023-41360-0 37696809 PMC10495370

[B22] Jones J. D. G. Dangl J. L. (2006). The plant immune system. Nature 444, 323–329. doi: 10.1038/nature05286 17108957

[B23] Kim S.-K. Rajapakse N. (2005). Enzymatic production and biological activities of chitosan oligosaccharides (COS): A review. Carbohydr. Polymers 62, 357–368. doi: 10.1016/j.carbpol.2005.08.012

[B24] Lecourieux D. Ranjeva R. Pugin A. (2006). Calcium in plant defence-signalling pathways. New Phytol. 171, 249–269. doi: 10.1111/j.1469-8137.2006.01777.x 16866934

[B25] Li H. Guan Y. Dong Y. Zhao L. Rong S. Chen W.. (2018a). Isolation and evaluation of endophytic *Bacillus tequilensis GYLH001* with potential application for biological control of Magnaporthe oryzae. PloS One 13, e0203505. doi: 10.1371/journal.pone.0203505 30379821 PMC6209128

[B26] Li J. Wang X. Lin X. Yan G. Liu L. Zheng H.. (2018b). Alginate-derived oligosaccharides promote water stress tolerance in *cucumber (Cucumis sativus L.)* . Plant Physiol. Biochem. 130, 80–88. doi: 10.1016/j.plaphy.2018.06.040 29980096

[B27] Liu T. Liu Z. Song C. Hu Y. Han Z. She J.. (2012). Chitin-induced dimerization activates a plant immune receptor. Science 336, 1160–1164. doi: 10.1126/science.1218867 22654057

[B28] Liu S. Wang J. Han Z. Gong X. Zhang H. Chai J. (2016). Molecular mechanism for fungal cell wall recognition by *rice* chitin receptor osCEBiP. Structure 24, 1192–1200. doi: 10.1016/j.str.2016.04.014 27238968

[B29] Liu H. Zhang Y.-H. Yin H. Yin H. Wang W.-X. Zhao X.-M.. (2013). Alginate oligosaccharides enhanced *Triticum aestivum L.* tolerance to drought stress. Plant Physiol. Biochem. 62, 33–40. doi: 10.1016/j.plaphy.2012.10.012 23178482

[B30] Lozowicka B. Abzeitova E. Sagitov A. Kaczynski P. Toleubayev K. Li A. (2015). Studies of pesticide residues in *tomatoes* and *cucumbers* from Kazakhstan and the associated health risks. Environ. Monit Assess. 187, 609. doi: 10.1007/s10661-015-4818-6 26337756 PMC4559566

[B31] Ma L. J. Li X. M. Bu N. Li N. (2010). An alginate-derived oligosaccharide enhanced *wheat* tolerance to cadmium stress. Plant Growth Regul. 62, 71–76. doi: 10.1007/s10725-010-9489-2

[B32] Miya A. Albert P. Shinya T. Desaki Y. Ichimura K. Shirasu K.. (2007). CERK1, a LysM receptor kinase, is essential for chitin elicitor signaling in *Arabidopsis* . Proc. Natl. Acad. Sci. U.S.A. 104, 19613–19618. doi: 10.1073/pnas.0705147104 18042724 PMC2148337

[B33] Natsume M. Kamo Y. Hirayama M. Adachi T. (1994). Isolation and characterization of alginate-derived oligosaccharides with root growth-promoting activities. Carbohydr Res. 258, 187–197. doi: 10.1016/0008-6215(94)84085-7 8039175

[B34] Nisa H. Kamili A. N. Nawchoo I. A. Shafi S. Shameem N. Bandh S. A. (2015). Fungal endophytes as prolific source of phytochemicals and other bioactive natural products: A review. Microb. Pathog. 82, 50–59. doi: 10.1016/j.micpath.2015.04.001 25865953

[B35] Peng C. Wang Q. Jiao R. Xu Y. Han N. Wang W.. (2021). A novel chondroitin sulfate E from Dosidicus gigas cartilage and its antitumor metastatic activity. Carbohydr Polym 262, 117971. doi: 10.1016/j.carbpol.2021.117971 33838835

[B36] Peng C. Wang Q. Lu D. Han W. Li F. (2018). A novel bifunctional endolytic alginate lyase with variable alginate-degrading modes and versatile monosaccharide-producing properties. Front. Microbiol. 9. doi: 10.3389/fmicb.2018.00167 PMC5809466 29472911

[B37] Peng C. Zhang A. Wang Q. Song Y. Zhang M. Ding X.. (2020). Ultrahigh-activity immune inducer from Endophytic Fungi induces *tobacco* resistance to virus by SA pathway and RNA silencing. BMC Plant Biol. 20, 169. doi: 10.1186/s12870-020-02386-4 32293278 PMC7160901

[B38] Qin F. Gao Y. X. Guo B. Y. Xu P. Li J. Z. Wang H. L. (2014). Environmental behavior of benalaxyl and furalaxyl enantiomers in agricultural soils. J. Environ. Sci. Health B 49, 738–746. doi: 10.1080/03601234.2014.929482 25065825

[B39] Rodriguez-Salus M. Bektas Y. Schroeder M. Knoth C. Vu T. Roberts P.. (2016). The synthetic elicitor 2-(5-bromo-2-hydroxy-phenyl)-thiazolidine-4-carboxylic acid links plant immunity to hormesis1. Plant Physiol. 170, 444–458. doi: 10.1104/pp.15.01058 26530314 PMC4704575

[B40] Salachna P. Grzeszczuk M. Meller E. Soból M. (2018). Oligo-Alginate with Low Molecular Mass Improves Growth and Physiological Activity of *Eucomis autumnalis* under Salinity Stress. Molecules 23, 812. doi: 10.3390/molecules23040812 29614824 PMC6017372

[B41] Shinya T. Motoyama N. Ikeda A. Wada M. Kamiya K. Hayafune M.. (2012). Functional characterization of CEBiP and CERK1 homologs in *arabidopsis* and *rice* reveals the presence of different chitin receptor systems in plants. Plant Cell Physiol. 53, 1696–1706. doi: 10.1093/pcp/pcs113 22891159

[B42] Shinya T. Nakagawa T. Kaku H. Shibuya N. (2015). Chitin-mediated plant-fungal interactions: catching, hiding and handshaking. Curr. Opin. Plant Biol. 26, 64–71. doi: 10.1016/j.pbi.2015.05.032 26116978

[B43] Sticher L. Mauch-Mani B. Métraux J. P. (1997). Systemic acquired resistance. Annu. Rev. Phytopathol. 35, 235–270. doi: 10.1146/annurev.phyto.35.1.235 15012523

[B44] Sun Y. Detchemendy T. W. Pajerowska-Mukhtar K. M. Mukhtar M. S. (2018). NPR1 in jazzSet with pathogen effectors. Trends Plant Sci. 23, 469–472. doi: 10.1016/j.tplants.2018.04.007 29753632

[B45] Tang J. Zhou Q. Chu H. Nagata S. (2011). Characterization of alginase and elicitor-active oligosaccharides from Gracilibacillus A7 in alleviating salt stress for *Brassica campestris L* . J. Agric. Food Chem. 59, 7896–7901. doi: 10.1021/jf201793s 21696216

[B46] Tomoda Y. Umemura K. Adachi T. (1994). Promotion of *barley* root elongation under hypoxic conditions by alginate lyase-lysate (A.L.L.). Biosci. Biotechnol. Biochem. 58, 202–203. doi: 10.1271/bbb.58.202 27315724

[B47] Ton J. Flors V. Mauch-Mani B. (2009). The multifaceted role of ABA in disease resistance. Trends Plant Sci. 14, 310–317. doi: 10.1016/j.tplants.2009.03.006 19443266

[B48] Van Wees S. C. M. van der Ent S. Pieterse C. M. J. (2008). Plant immune responses triggered by beneficial microbes. Curr. Opin. Plant Biol. 11, 443–448. doi: 10.1016/j.pbi.2008.05.005 18585955

[B49] Wiesel L. Newton A. C. Elliott I. Booty D. Gilroy E. M. Birch P. R. J.. (2014). Molecular effects of resistance elicitors from biological origin and their potential for crop protection. Front. Plant Sci. 5. doi: 10.3389/fpls.2014.00655 PMC4240061 25484886

[B50] Xing M. Cao Q. Wang Y. Xiao H. Zhao J. Zhang Q.. (2020). Advances in research on the bioactivity of alginate oligosaccharides. Mar. Drugs 18, 144. doi: 10.3390/md18030144 32121067 PMC7142810

[B51] Xu X. Iwamoto Y. Kitamura Y. Oda T. Muramatsu T. (2003). Root growth-promoting activity of unsaturated oligomeric uronates from alginate on *carrot* and *rice* plants. Biosci Biotechnol Biochem. 67, 2022–2025. doi: 10.1271/bbb.67.2022 14519996

[B52] Xue N. Yang R. Xu X. Seip H. M. Zeng Q. (2006). Adsorption and degradation of benfuracarb in three soils in Hunan, People’s Republic of China. Bull. Environ. Contam Toxicol. 76, 720–727. doi: 10.1007/s00128-006-0979-x 16688558

[B53] Yadav I. C. Devi N. L. Syed J. H. Cheng Z. Li J. Zhang G.. (2015). Current status of persistent organic pesticides residues in air, water, and soil, and their possible effect on neighboring countries: a comprehensive review of India. Sci. Total Environ. 511, 123–137. doi: 10.1016/j.scitotenv.2014.12.041 25540847

[B54] Yang K. Y. Liu Y. Zhang S. (2001). Activation of a mitogen-activated protein kinase pathway is involved in disease resistance in *tobacco* . Proc. Natl. Acad. Sci. United States America 98, 741–746. doi: 10.1073/pnas.98.2.741 PMC14658 11209069

[B55] Yang C. Wang E. Liu J. (2022). CERK1, more than a co-receptor in plant-microbe interactions. New Phytol. 234, 1606–1613. doi: 10.1111/nph.18074 35297054

[B56] Yin H. Du Y. Dong Z. (2016). Chitin oligosaccharide and chitosan oligosaccharide: two similar but different plant elicitors. Front. Plant Sci. 7. doi: 10.3389/fpls.2016.00522 PMC4840203 27148339

[B57] Yonemoto Y. Tanaka H. Yamashita T. Kitabatake N. Ishida Y. Kimura A.. (1993). Promotion of germination and shoot elongation of some plants by alginate oligomers prepared with bacterial alginate lyase. J. Fermentation Bioengineering 75, 68–70. doi: 10.1016/0922-338X(93)90181-7

[B58] Zang H. Xie S. Zhu B. Yang X. Gu C. Hu B.. (2019). Mannan oligosaccharides trigger multiple defence responses in *rice* and *tobacco* as a novel danger-associated molecular pattern. Mol. Plant Pathol. 20, 1067–1079. doi: 10.1111/mpp.12811 31094073 PMC6640537

[B59] Zhang H. Fang Q. Zhang Z. Wang Y. Zheng X. (2009). The role of respiratory burst oxidase homologues in elicitor-induced stomatal closure and hypersensitive response in *Nicotiana benthamiana* . J. Exp. Bot. 60, 3109–3122. doi: 10.1093/jxb/erp146 19454596 PMC2718215

[B60] Zhang C. Howlader P. Liu T. Sun X. Jia X. Zhao X.. (2019). Alginate Oligosaccharide (AOS) induced resistance to Pst DC3000 via salicylic acid-mediated signaling pathway in *Arabidopsis thaliana* . Carbohydr Polym 225, 115221. doi: 10.1016/j.carbpol.2019.115221 31521273

[B61] Zhang Y. Yin H. Zhao X. Wang W. Du Y. He A.. (2014). The promoting effects of alginate oligosaccharides on root development in *Oryza sativa L.* mediated by auxin signaling. Carbohydr Polym 113, 446–454. doi: 10.1016/j.carbpol.2014.06.079 25256506

[B62] Zipfel C. Kunze G. Chinchilla D. Caniard A. Jones J. D. G. Boller T.. (2006). Perception of the bacterial PAMP EF-tu by the receptor EFR restricts agrobacterium-mediated transformation. Cell 125, 749–760. doi: 10.1016/j.cell.2006.03.037 16713565

