# Supplementary material for: Alginate oligosaccharides trigger multiple defense responses in tobacco and induce resistance to Phytophthora infestans
Source: Front Plant Sci. 2025 Feb 12;16:1506873. doi: 10.3389/fpls.2025.1506873 (PMC11863610; doi:10.3389/fpls.2025.1506873)
Supplement: Supplementary file 1 [file DataSheet1.docx]

**Supporting information**

Additional Supporting Information may be found in the online version of this article.

**Figure S1.** AOS induces callose accumulation in *N*. *benthamiana* (A) Variation of callose fluorescence intensity in Arabidopsis thaliana; (B) Quantitative analysis of callose fluorescence intensity. Results are presented as the average fluorescence intensity of leaves tissue using ZEN software. The experiments were repeated three times. Error bars indicate SEM. Statistics by Student’s t-test (* P≤0.05，**P≤0.01).

**Figure S2.**  AtCERK1 is a key receptor kinase when AOS induces resistance to pathogens. (A) *Arabidopsis* mutants and wild-type *Arabidopsis* leaves were inoculated with *P*. *infestas*; (B) Relative expression levels of the *AtCEBiP-LIKE1* and *AtCERK1* genes after AOS treatment for 24 h.


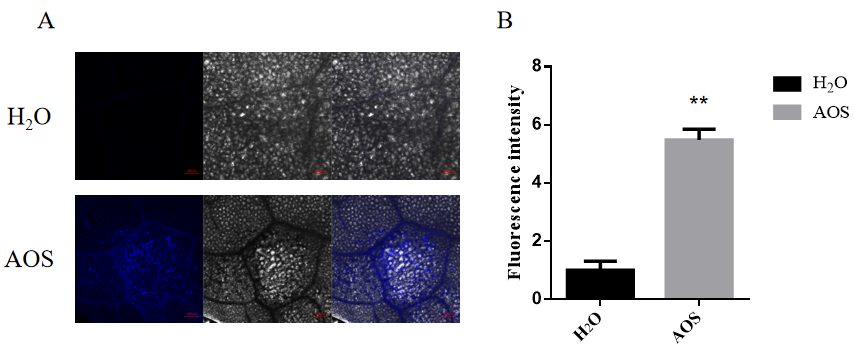


Figure S1


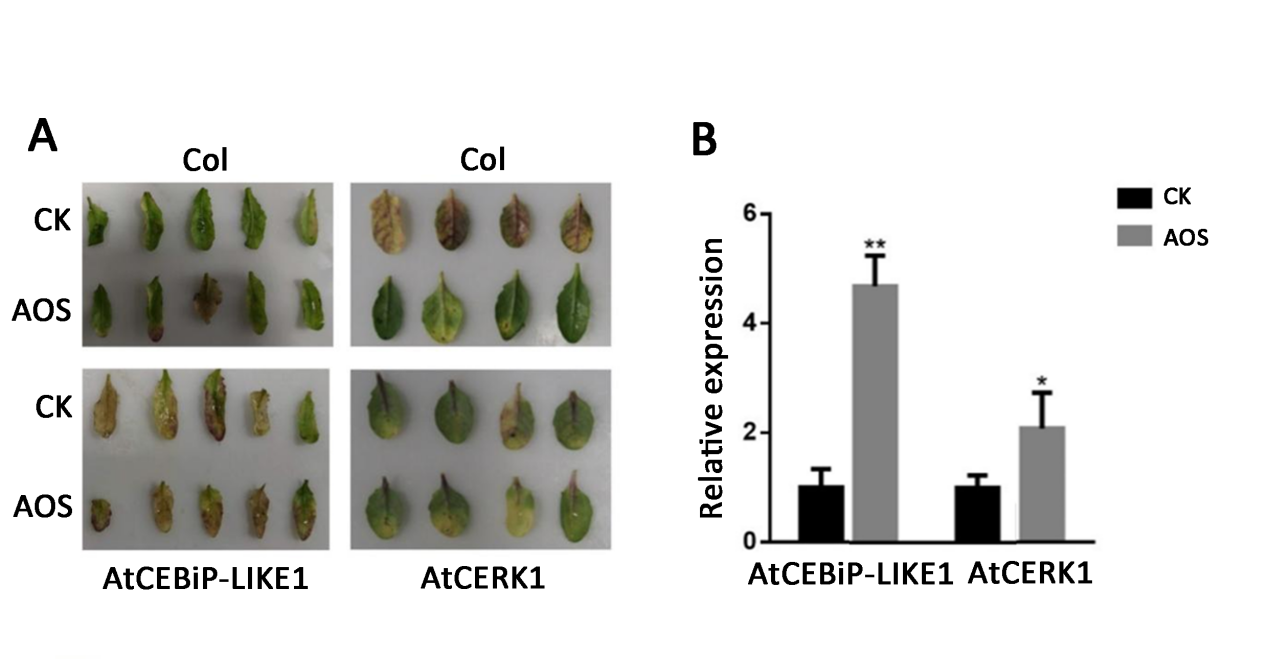


Figure S2
